# Factors Affecting Rapid Cognitive Decline in Patients with Alzheimer’s Disease: A Longitudinal Follow-Up Study

**DOI:** 10.3390/ijerph18168576

**Published:** 2021-08-13

**Authors:** Chih-Chuan Pan, Che-Sheng Chu, Chien-Liang Chen, Yao-Chung Chuang, Nai-Ching Chen

**Affiliations:** 1 Department of Psychiatry, Kaohsiung Veterans General Hospital, Kaohsiung 813, Taiwan; ccpan@vghks.gov.tw (C.-C.P.); youngtzuchi@hotmail.com (C.-S.C.); 2 Center for Geriatric and Gerontology, Kaohsiung Veterans General Hospital, Kaohsiung 813, Taiwan; 3 Non-Invasive Neuromodulation Consortium for Mental Disorders, Society of Psychophysiology, Taipei 114, Taiwan; 4 Graduate Institute of Medicine, College of Medicine, Kaohsiung Medical University, Kaohsiung 807, Taiwan; 5 Division of Nephrology, Kaohsiung Veterans General Hospital, Kaohsiung 813, Taiwan; cclchen@seed.net.tw; 6 Department of Medicine, National Yang Ming Chiao Tung University, Taipei 112, Taiwan; 7 Department of Neurology, Kaohsiung Chang Gung Memorial Hospital, Kaohsiung 833, Taiwan; ycchuang@cgmh.org.tw; 8 Department of Neurology, School of Medicine, College of Medicine, Kaohsiung Medical University, Kaohsiung 807, Taiwan

**Keywords:** acetylcholinesterase inhibitors, Alzheimer’s disease, rapid cognitive decline, predictor, moderators

## Abstract

We investigated the preventive and risk factors of rapid cognitive decline in patients with Alzheimer’s disease (AD). Using the Chang Gung Research Database (CGRD), we enrolled patients with AD aged over 65 years between 1 January 2001 and 30 May 2019, and followed up for at least two years. Rapid cognitive decline was defined by a Mini-Mental State Examination (MMSE) score decline of ≥4 in 2 years. A longer prescription of acetylcholinesterase inhibitors (AChEIs) was defined as 22 months based on the median treatment duration of the cohorts. The Cox proportional hazards regression model adjusted for age, sex, medication, and physical comorbidities was used to examine the candidate risk and protective factors. We analyzed data from 3846 patients with AD (1503 men, 2343 women) with a mean age and percentage of females of 77.8 ± 6.2 years and 60.9%, respectively. The mean duration of patients with AD receiving AChEIs was 658.7 ± 21.9 days. In general, 310 patients with AD showed a rapid cognitive decline, accounting for 8.1%. Treatment of a consecutive AChEI prescription for >22 months in patients with AD was a protective factor against rapid cognitive decline (adjusted hazard ratio (aHR) = 0.41, 95% confidence interval (CI) = 0.33–0.52, *p* < 0.001). Patients with AD aged >85 years (aHR = 0.53, 95% CI = 0.36–0.79, *p* < 0.01) and aged 75–85 years (aHR = 0.73, 95% CI = 0.57–0.93, *p* < 0.05) had a significantly lower risk of rapid cognitive decline than those aged 65–75 years. Additionally, patients with mild and moderate AD (clinical dementia rating (CDR = 1, aHR = 1.61, 95% CI = 1.26–2.07, *p* < 0.001; CDR = 2, aHR = 2.64, 95% CI = 1.90–3.65, *p* < 0.001) were more likely to have rapid cognitive decline than those with early AD (CDR = 0.5). Sex, medication with different types of AChEIs, and physical comorbidities were not associated with rapid cognitive decline. These findings indicate that it is important to maintain longer consecutive AChEI prescriptions in patients with AD to prevent cognitive decline.

## 1. Introduction

Alzheimer’s disease (AD), a progressive neurodegenerative disease, is the most common form of dementia and is characterized by a gradual decline in cognition (mainly memory), behavioral and social skills, and daily function [1]. With the global growth in the aging population, increasing numbers of patients with dementia will place a burden on the patients themselves, caregivers, and healthcare systems [2]. On 7 June 2021, the Food and Drug Administration of the United States granted accelerated approval to aducanumab, the first anti-amyloid and disease modifying therapy, for treatment of AD. However, the approval has sparked controversy among experts [3].

To date, most established treatments are symptomatic in nature, including acetylcholinesterase inhibitors (AChEIs) (rivastigmine, galantamine, and donepezil) and memantine, an ^N^-methyl-d-aspartate (NMDA) receptor antagonist [4]. Although these drugs can only provide slight cognitive benefits, both AChEIs and memantine are still most commonly used to delay the progression of cognitive dysfunction and improve other symptoms in patients with AD [4,5,6,7]. Several randomized controlled trials (RCTs) showed sustained use of AChEIs for 3 to 12 months was reported to delay the progressive cognitive, functional, and behavioral degeneration caused by AD [5,6,7].

Patients with AD show gradual and continued cognitive and functional decline over the course of the disease [1]. However, the rate of progression varies among individuals, partly because of different conceptual definitions across different studies [8]. Generally, it can be divided into rapid and non-rapid decline according to the mean decline of 2–3 Mini-Mental State Examination (MMSE) points/year [9]. Patients with rapid cognitive decline tend to have a worse prognosis regarding function and mortality [2]. Several studies have attempted to identify factors associated with rapid decline for early detection of cognitive change [10,11,12] and provide prompt intervention for both clinicians and caregivers. RCTs are optimally designed to examine the efficacy of AChEIs on cognition but tend to have inadequate power to detect individual variability in response due to relatively small samples and substantial attrition [13]. Restricting trial inclusion and exclusion criteria may limit the generalizability of the findings to “real-world” clinical settings.

The rate of clinical decline seems to vary based on different stages of AD [11,14,15], age at the start of AChEI treatment [11,15], and comorbidity with hypertension (HTN) [10,16] and type 2 diabetes mellitus (T2DM) [12,17]. These factors may influence the therapeutic effectiveness of AChEIs in disease progression in patients with AD. However, previous studies used RCT design to demonstrate the efficacy of AChEIs on cognition for up to one year [5,6,7], providing inadequate follow-up time to detect cognitive decline. Therefore, the present study used real-world data to include large sample sizes and investigated the efficacy of AChEIs (donepezil, rivastigmine, and galantamine) on cognitive decline and examined the possible moderators.

## 2. Materials and Methods

### 2.1. Data Source

The Chang Gung Medical Foundation (CGMF) is the largest medical system in Taiwan. Electronic medical records derived from Chang Gung Memorial Hospital (CGMH) to comprise the Chang Gung Research Database (CGRD) are used to provide real-world evidence and improve clinical and policy decisions [18].

### 2.2. Selection of Patients with AD and Comorbidities

Using the CGRD, we enrolled patients aged ≥65 years who were diagnosed with AD (ICD-9-CM codes: 290.0−290.3 and 331.0) by certified psychologists or well-trained neurologists, based on strict reviews of medical records, brain images, and blood and cognitive test results, between 1 January 2001 and 30 May 2019. AD patients were required to maintain the same AChEIs (donepezil, rivastigmine, and galantamine) for at least 1 year, and undergo the Mini-Mental State Examination (MMSE) and clinical dementia rating scale (CDR) examination at least twice before 2017 to allow two-year follow-up. We excluded patients with AD during the follow-up period, patients who switched AChEIs, patients for whom MMSE or CDR were unavailable, and advanced AD patients with CDR 3–5. Rapid cognitive decline was identified during the follow-up period (from enrollment to 30 December 2019). Rapid decline was defined as an MMSE score decline of ≥4 in 2 years based on a previous study [19] and the National Health Insurance Bureau of Taiwan, which denied prescription AChEIs during the following year for those with an MMSE decline of >2 in a single year. Physical comorbidities included pulmonary disease, renal disease, liver disease, T2DM, hypertension, and hyperlipidemia.

### 2.3. Patient Data and Their Anonymity

To ensure data privacy, patient and provider information was encrypted and de-identified [18]. The participants signed a declaration for the protection of computer-processed personal data and to take legal responsibility based on Taiwan’s Personal Information Protection Act. Furthermore, the study protocol was approved by the Ethics Committee of the Institutional review Board of CGMH. This study was conducted in accordance with the tenets of the Declaration of Helsinki and was approved by the Institutional Review Board (IRB No. 201900866B0). Informed consent was waived according to IRB regulations.

### 2.4. Statistical Analysis

All data processing and statistical analyses were performed using SAS version 9.4. (SAS Institute Inc., Cary, NC, USA). Descriptive statistics are expressed as mean ± standard deviation or number (percentage). The between-group differences were compared using the Student’s *t*-test with a normal distribution. Chi-square tests were used to analyze categorical variables, including sex, types of AChEI, CDR, and comorbidities. The Cox proportional hazards regression model was adjusted for age, sex, medication, and physical comorbidities to calculate hazard ratios (HRs) with a 95% confidence interval (CI) between rapid cognitive decline and non-rapid cognitive decline cohorts. Sensitivity analysis was performed to clarify the independent role of different stages of AD, age at the start of AChEI treatment, and comorbidity with HTN and T2DM. Two-sided values of *p* < 0.05 were considered statistically significant.

## 3. Results

### 3.1. Characteristics of Study Participants

**Figure 1:**
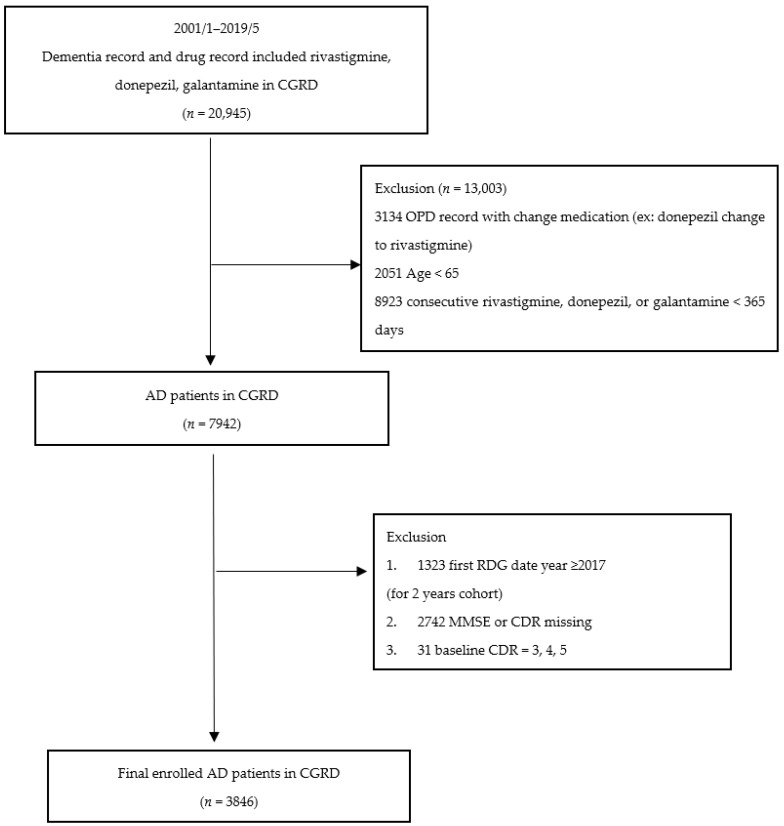
Flowchart of selection of study subjects.

Figure 1 shows the flowchart for the selection of the study subjects. Table 1 lists the sociodemographic and clinical data of the patients with AD. Data from 3846 patients with AD (1503 men and 2343 women) were analyzed. The mean age of the entire sample and percentage of females were 77.8 ± 6.2 years and 60.9%, respectively. The mean duration of patients with AD receiving AChEIs was 658.7 ± 21.9 days. The consecutive AChEI prescription of longer vs. shorter treatment duration, defined as 22 months, was based on the median drug prescription duration of the whole study population. The baseline MMSE before receiving AChEIs was 17.2 ± 5.3 and the score declined to 15.8 ± 6.3 following 2 year follow-up. In general, 310 patients with AD showed rapid cognitive decline (decline >3 scores of MMSE in 2 years), accounting for 8.1%.

### 3.2. Risk of Rapid Cognitive Decline among Patients with AD

As shown in Table 2, for patients with AD, receipt of consecutive AChEI prescriptions for >22 months was a protective factor against rapid cognitive decline (aHR = 0.41, 95% CI = 0.33–0.52, *p* < 0.001) compared to those with prescription ≤22 months. Patients with AD aged >85 years (aHR = 0.53, 95% CI = 0.36–0.79, *p* < 0.01) and aged 75–85 years (aHR = 0.73, 95% CI = 0.57–0.93, *p* < 0.05) had a significantly lower risk of rapid cognitive decline than those aged 65–75 years. Additionally, patients with mild to moderate AD (CDR = 1, aHR = 1.61, 95% CI = 1.26–2.07, *p* < 0.001; CDR = 2, aHR = 2.64, 95% CI = 1.90–3.65, *p* < 0.001) were more likely to have rapid cognitive decline than those with early AD (CDR = 0.5). Sex, medication with different types of AChEI, and physical comorbidities were not associated with rapid cognitive decline.

### 3.3. Sensitivity Analysis: Risk of Rapid Cognitive Decline among Patients with AD Stratified by Age

Table 3 shows that, in patients with AD, receiving consecutive AChEI prescriptions for >22 months was a protective factor against rapid cognitive decline across different age distributions (aged 65–75, aHR = 0.37, 95% CI = 0.25–0.54, *p* < 0.001; aged 76–85, aHR = 0.47, 95% CI = 0.34–0.66, *p* < 0.001; aged >85, aHR = 0.25, 95% CI = 0.12–0.53, *p* < 0.001) after adjustment compared to those with prescriptions for ≤22 months. Among patients aged 65–75 years, patients with mild and moderate AD were 2.06-fold and 2.85-fold more likely, respectively, to have rapid cognitive decline than those with early AD. Among those aged 76–85 years and >85 years, patients with moderate AD were 2.57-fold and 3.71-fold more likely, respectively, to have rapid cognitive decline than those with early AD, but no increased risk was found for patients with mild AD. Female patients with AD aged >85 years were less likely to have rapid cognitive decline than male patients (aHR = 0.25, 95% CI = 0.12–0.53, *p* < 0.001). We did not find a gender effect in patients with AD aged 65–75 years and 76–85 years.

### 3.4. Sensitivity Analysis: Risk of Rapid Cognitive Decline among Patients with AD Stratified by HTN and T2DM

As shown in Table 4, for patients with AD, receiving consecutive AChEI prescriptions for >22 months was a protective factor against rapid cognitive decline among those comorbid with and without T2DM (with T2DM, aHR = 0.54, 95% CI = 0.33–0.90, *p* < 0.05; without T2DM, aHR = 0.37, 95% CI = 0.29–0.49, *p* < 0.001) after adjustment compared to those with less than 22 months of prescription. Among AD patients without T2DM, the risk of rapid cognitive decline for those aged >85 years and 76-85 years were significantly reduced by 56% and 31% compared to those aged 65–75 years, whereas no reduced risk was found for patients with T2DM. Furthermore, mild and moderate AD patients without T2DM were 1.51-fold and 2.95-fold more likely to have rapid cognitive decline than those with early AD, whereas the increased risk only existed in patients with mild AD and T2DM (aHR = 2.09, 95% CI = 1.26–3.49, *p* < 0.01) rather than those with moderate AD.

As shown in Table 5, for patients with AD, receiving consecutive AChEI prescriptions for >22 months was a protective factor against rapid cognitive decline among those comorbid with and without HTN (with HTN, aHR = 0.46, 95% CI = 0.33–0.63, *p* < 0.001; without HTN, aHR = 0.35, 95% CI = 0.25–0.50, *p* < 0.001) after adjustment compared to those with less than 22 months of prescription. Among AD patients with HTN, the risk of rapid cognitive decline for those aged >85 years was significantly reduced, by 49%, compared to those aged 65–75 years, whereas a reduction in risk of 30% was found in AD patients without HTN aged 76–85. Furthermore, mild and moderate AD patients with HTN were 1.95-fold and 3.55-fold more likely to have rapid cognitive decline than those with early AD, whereas the increased risk (1.84-fold) only existed in moderate AD patients without HTN, and not in those with early AD. Finally, among AD patients without HTN, those with comorbid renal disease were 2.94-fold more likely to have rapid cognitive decline than those without renal disease, whereas renal disease did not influence the rate of cognitive decline in the subgroup of AD patients with HTN.

## 4. Discussion

This study was derived from routine clinical practice, thus demonstrating its ecological validity. The findings of the present study were as follows: (1) AD patients receiving longer consecutive AChEI prescriptions had a significantly reduced risk of rapid cognitive decline than those with shorter prescriptions. The protective effect persisted across sensitivity analysis stratified by age distribution, and comorbid T2DM and HTN. (2) AD patients with greater overall severity measured by CDR and with younger age at enrollment were significantly more likely to have rapid cognitive decline. (3) The subgroup of more elderly (aged >85 years) AD female patients had a significantly lower risk of rapid cognitive decline than male patients, whereas the gender effect was not found in those aged 65–75 and 76–85 years. (4) The subgroup of AD patients without T2DM, aged >85 and 76–85 years, received a protective effect against rapid cognitive decline compared to those aged 65–75 years, whereas the age effect was not found in AD patients with T2DM. (5) Within the subgroup of AD patients without HTN, those with comorbid renal disease were more likely to have rapid cognitive decline than those without renal disease, whereas the renal disease did not impact the progression in the subgroup of AD patients with HTN. (6) No association was found between the type of medication (donepezil, rivastigmine, and galantamine), comorbidities (HTN and DM), and the rate of progression.

The main finding of this study is that longer consecutive AChEI prescriptions significantly reduced the risk of rapid cognitive decline compared to those with shorter prescriptions. This is consistent with previous RCTs of 3 to 12 months [5,6,7]. These durations limit the studies’ generalizability and external validity because they do not always reflect clinical situations. The study confirmed the beneficial results of AChEIs based on real-world claims of large sample sizes and extended the follow-up period to 2 years. We also showed that patients with more severe AD according to CDR were prone to rapid progression. AD patients with mild (CDR = 1) and moderate (CDR = 2) severity were 1.61- and 2.64-fold more likely to show rapid cognitive decline compared to those with a CDR of 0.5. This finding is consistent with several studies [11,14,15], showing that AD patients who are more severely impaired in the early stages will decline faster than less impaired patients. Regarding various AChEIs (donepezil, rivastigmine, and galantamine), the present study demonstrated no obvious difference in the therapeutic response assessed. The findings of equal efficacy of AChEIs on cognition from real-word datasets were in line with previous head-to-head trials [20] and meta-analysis studies [21].

Age may influence the rate of cognitive decline; however, this effect is mixed. Younger AD patients have a more rapid cognitive decline [22,23], partly due to the more pathological burden of neuritic plaques seen in younger patients with AD than in older patients with AD [24]. Other studies have demonstrated that older age contributes to a more significant decline in cognition [11,15]. However, older age at baseline was associated with a slower decline in a pooled cohort study of patients with AD [25], which is consistent with the present study. We found that individuals aged >85 years and aged 76–85 years were less likely to have rapid cognitive decline, by 47% and 37%, respectively, compared to those aged 65–75 years. The present study included an older population (mean age, approximately 78 years) with a survival bias of resilience to cognitive decline [26], which may explain the conflicting results. Finally, female AD patients aged over 85 years had a significantly reduced risk of rapid cognitive decline compared to male patients, which is consistent with a previous study [27,28]. The better cognitive function in females may be partly due to the relative freedom from cardiovascular disease in females [28].

T2DM is a well-established risk factor for AD. However, several studies have found that AD patients with T2DM are less likely to experience cognitive decline compared to those without T2DM [12,17,29], demonstrating that AD patients without T2DM are at risk for cognitive progression. The present study showed no impact of comorbid T2DM on disease progression. Furthermore, few studies have examined the effect of AChEIs on the rate of progression between AD patients with and without T2DM. We found that AD patients without T2DM receiving longer AChEIs showed a 63% reduction in rapid cognitive decline compared to those with shorter AChEIs; the protective effect was reduced by 46% in AD patients with T2DM. A nationwide claim dataset found inadequate clinical management with fewer AChEI prescriptions for AD patients with T2DM compared to those without T2DM [30], which may explain the difference. Finally, AD patients without T2DM have a significantly reduced risk of rapid decline across different age distributions, whereas the effect disappears among AD patients with T2DM divided by different age distributions. The age-protective effect differs among subgroups of patients with AD, with and without T2DM. Future studies should explore the underlying mechanisms.

Several studies have examined the association between comorbid HTN and the risk of disease progression in patients with AD, but the results are inconsistent. Some studies have reported that HTN predicts rapid decline [16,31,32], whereas others showed no risk [10,17,33]. The present study found that comorbid HTN in patients with AD did not impact disease progression. Furthermore, moderate AD (CDR = 2) with HTN contributed a higher risk of rapid decline compared to moderate AD alone, with a 3.55-fold higher risk compared to a 1.84-fold higher risk. HTN contributes to cognitive decline via demyelination or microinfarction of cerebral white matter [34]; therefore, the additive effects of HTN on AD pathology may, at least in part, contribute to a higher risk of rapid decline. Another interesting finding was that in the subgroup of patients with AD without HTN, those with comorbid renal disease had a 2.94-fold higher risk of rapid decline compared to those without renal disease, whereas the association was not found in the subgroup of patients with AD and HTN. Patients with cognitive impairment tend to suffer from orthostatic hypotension and age-related arteriosclerotic changes, resulting in vulnerability to cerebral hypoperfusion [35]. The associated cerebral hypoperfusion may be more frequent in patients with AD without HTN than in those with HTN. However, it is not easy to provide a detailed explanation, and the issue is beyond the scope of the present study. Further studies are warranted to explore the possible mechanisms and validate our findings.

### Strengthens and Limitations

The strengths of this study are the use of the CGRD. This provides more clinical information, particularly for cognitive evaluation results, which is unavailable in the National Health Insurance Research Database in Taiwan. Therefore, this allowed us to examine the potential moderators of rapid cognitive decline in a large sample of patients with AD. In Taiwan, the diagnosis of AD can only be established by certified psychologists or well-trained neurologists based on strict reviews of medical records, brain images, and blood and cognitive test results. After receiving certification permission, the patients were eligible to receive AChEIs. Therefore, the validation of the accurate diagnosis of AD is better than that of other claim datasets, which are mainly based on ICD-9-CM or the *Diagnostic and Statistical Manual of Mental Disorders* criteria [36].

However, this study has several limitations. First, the patients’ demographic data from the CGRD may differ from those of the national database, with the CGRD presenting more elderly outpatients and a higher severity of comorbidities [37]. However, we adjusted for several confounders and conducted sensitivity analysis based on different age groups, and comorbid T2DM and HTN, to address this issue. Second, the CGRD data collected before 2000 were traditional paper medical records, which are not currently available for researchers. Third, patients’ medical information in the CGRD relating to visits to other hospitals for any medical issues will be not available; therefore, the information from outside visits does not appear in the data. Fourth, we did not assess the impact of medications other than AChEIs (e.g., anti-hypertension drugs and statins) on progression because these may influence cognitive function [38]. However, this was beyond the scope of the present study.

## 5. Conclusions

The present study showed that longer consecutive AChEI prescriptions were associated with a slower rate of cognitive decline compared to short prescriptions. Thus, it is important to maintain adequate treatment to prevent cognitive decline in patients with AD.

## Figures and Tables

**Table 1:** Demographic and clinic characteristics of patients with Alzheimer’s disease.

Variables	Mean ± SD or Percentage (%)
Patients, number	3846
Age	77.8 ± 6.2
Gender	
Male	1503 (39.1)
Female	2343 (60.9)
Physical comorbidities	
Pulmonary disease	386 (10.0)
Renal disease	366 (9.5)
Liver disease	317 (8.2)
T2DM	931 (24.2)
HTN	2035 (52.9)
Hyperlipidemia	1142 (29.7)
Medication	
Rivastigmine	1833 (47.7)
Donepezil	1736 (45.1)
Galantamine	277 (7.2)
Duration of AChEI use days	658.7 ± 21.9
Consecutive AChEI Prescription	
≤22 months	1949 (50.7)
>22 months	1897 (49.3)
Baseline cognitive test before AChEIs	
MMSE	17.2 ± 5.3
CDR	
0.5	2281 (59.3)
1	1196 (31.1)
2	369 (9.6)
Cognitive test following 2 years follow-up	
MMSE	15.8 ± 6.3
CDR	
0.5	1561 (40.6)
1	1558 (40.5)
2	639 (16.6)
≥3	88 (2.3)
Cognitive decline (defined by MMSE)	
Slow (decline ≤3 in 2 years), *n* (%)	3536 (91.9)
Rapid (decline >3 in 2 years), *n* (%)	310 (8.1)

Values are expressed as mean ± standard deviation (SD); numbers (percentages). Abbreviations: AChEIs, acetylcholinesterase inhibitors; CDR, clinical dementia rating scale; HTN, hypertension; MMSE, Mini-Mental State Examination; SD, standard deviation; T2DM, type 2 diabetic mellitus.

**Table 2:** Cox proportional hazards model for rapid decline within 2 years in patients with Alzheimer’s dementia.

	Adjusted HRs *	95% CI	*p* Value
Consecutive Drug Prescription			
≤22 months	Ref		
>22 months	0.41	0.33–0.52	**<0.001**
Age group			
65–75	Ref		
76–85	0.73	0.57–0.93	**<0.05**
>85M	0.53	0.36–0.79	**<0.01**
Gender			
Male	Ref		
Female	0.83	0.66–1.05	0.122
Medication			
Galantamine	-		
Rivastigmine	1.57	0.92–2.67	0.098
Donepezil	1.45	0.85–2.48	0.176
Baseline CDR			
0.5	Ref		
1	1.61	1.26–2.07	**<0.001**
2	2.64	1.90–3.65	**<0.001**
Comorbidity			
Pulmonary disease	1.22	0.86–1.73	0.267
Renal disease	1.11	0.74–1.65	0.616
Liver disease	0.85	0.55–1.30	0.446
T2DM	0.83	0.62–1.10	0.202
HTN	1.19	0.93–1.51	0.159
Hyperlipidemia	0.96	0.73–1.24	0.731

* Adjustment for age, sex, medication, and physical comorbidities. Bold type indicates statistical significance. Abbreviations: CDR, clinical dementia rating scale; CI, confidence interval; HRs, hazard ratios; HTN, hypertension; Ref, reference; T2DM, type 2 diabetic mellitus.

**Table 3:** Cox proportional hazards model for rapid decline within 2 years in patients with Alzheimer’s dementia in different age groups.

	Aged 65–75			Aged 76–85			Aged > 85		
	Adjusted HRs *	95% CI	*p* Value	Adjusted HRs *	95% CI	*p* Value	Adjusted HRs *	95% CI	*p* Value
Consecutive Drug Prescription									
≤22 month	Ref			Ref			Ref		
>22 months	0.37	0.25–0.54	**<0.001**	0.47	0.34–0.66	**<0.001**	0.25	0.12–0.53	**<0.001**
Gender									
Male	Ref			Ref			Ref		
Female	0.76	0.52–1.09	0.138	1.06	0.76–1.48	0.747	0.35	0.16–0.75	**<0.001**
Medication									
Galantamine	-			-			-		
Rivastigmine	1.50	0.64–3.48	0.350	1.40	0.67–2.91	0.369	2.49	0.32–19.59	0.387
Donepezil	1.06	0.45–2.50	0.898	1.44	0.69–3.00	0.333	4.64	0.60–36.06	0.143
Baseline CDR									
0.5	Ref			Ref			Ref		
1	2.06	1.40–3.03	**<0.001**	1.37	0.96–1.95	0.082	1.82	0.77–4.30	0.172
2	2.85	1.57–5.17	**<0.001**	2.57	1.66–3.97	**<0.001**	3.71	1.41–9.79	**<0.001**
Comorbidity									
Pulmonary diseas	1.45	0.84–2.51	0.183	1.33	0.79–2.21	0.281	0.55	0.19–1.63	0.284
Renal disease	1.09	0.57–2.05	0.800	1.17	0.65–2.10	0.607	1.17	0.37–3.75	0.790
Liver disease	1.36	0.74–2.50	0.326	0.54	0.28–1.08	0.081	0.59	0.12–2.81	0.509
T2DM	0.68	0.42–1.09	0.111	0.88	0.59–1.30	0.525	1.50	0.61–3.71	0.380
HTN	1.14	0.77–1.68	0.523	1.27	0.91–1.78	0.164	1.14	0.53–2.44	0.736
Hyperlipidemia	0.90	0.59–1.37	0.623	0.91	0.63–1.32	0.633	1.56	0.64–3.79	0.324

* Adjustment for age, sex, medication, and physical comorbidities. Bold type indicates statistical significance. Abbreviations: CDR, clinical dementia rating scale; CI, confidence interval; HRs, hazard ratios; HTN, hypertension; Ref, reference; T2DM, type 2 diabetic mellitus.

**Table 4:** Cox proportional hazards model for rapid decline within 2 years in patients with Alzheimer’s dementia with and without type 2 diabetes mellitus (DM).

	Patients with T2DM			Patients without T2DM		
	Adjusted HRs *	95% CI	*p* Value	Adjusted HRs *	95% CI	*p* Value
Consecutive Drug Prescription						
≤22 months	Ref			Ref		
>22 months	0.54	0.33–0.90	**<0.05**	0.37	0.29–0.49	**<0.001**
Age group						
65–75	Ref			Ref		
76–85	0.94	0.56–1.59	0.829	0.69	0.52–0.90	**<0.01**
>85	1.13	0.49–2.60	0.766	0.44	0.28–0.69	**<0.001**
Gender						
Male	Ref			Ref		
Female	0.74	0.45–1.20	0.220	0.87	0.67–1.13	0.297
Medication						
Galantamine	-			-		
Rivastigmine	1.78	0.55–5.80	0.336	1.50	0.83–2.73	0.181
Donepezil	1.27	0.38–4.25	0.698	1.47	0.81–2.68	0.206
Baseline CDR						
0.5	Ref			Ref		
1	2.09	1.26–3.49	**<0.01**	1.51	1.13–2.01	**<0.01**
2	1.44	0.61–3.42	0.404	2.95	2.07–4.20	**<0.001**
Comorbidity						
Pulmonary disease	0.70	0.29–1.65	0.412	1.37	0.93–2.02	0.107
Renal disease	0.88	0.47–1.68	0.709	1.25	0.75–2.06	0.392
Liver disease	0.99	0.48–2.01	0.967	0.78	0.45–1.34	0.368
HTN	0.91	0.52–1.60	0.752	1.25	0.96–1.63	0.093
Hyperlipidemia	1.06	0.65–1.73	0.805	0.90	0.66–1.24	0.523

* Adjustment for age, sex, medication, and physical comorbidities. Bold type indicates statistical significance. Abbreviations: CDR, clinical dementia rating scale; CI, confidence interval; HRs, hazard ratios; HTN, hypertension; Ref, reference; T2DM, type 2 diabetic mellitus.

**Table 5:** Cox proportional hazards model for rapid decline within 2 years in patients with Alzheimer’s dementia with hypertension and without hypertension.

	Patients with HTN			Patients without HTN		
	Adjusted HRs *	95% CI	*p* Value	Adjusted HRs *	95% CI	*p* Value
Consecutive Drug Prescription						
≤22 months	Ref			Ref		
>22 months	0.46	0.33–0.63	**<0.001**	0.35	0.25–0.50	**<0.001**
Age group						
65–75	Ref			Ref		
76–85	0.75	0.65–1.05	0.092	0.70	0.49–0.99	**<0.05**
>85	0.51	0.30–0.88	**<0.05**	0.57	0.32–1.03	0.064
Gender						
Male	Ref			Ref		
Female	0.79	0.57–1.08	0.138	0.91	0.65–1.28	0.607
Medication						
Galantamine	-			-		
Rivastigmine	0.99	0.51–1.93	0.987	2.48	0.98–6.34	0.050
Donepezil	0.92	0.47–1.79	0.800	2.30	0.92–5.75	0.074
Baseline CDR						
0.5	Ref			Ref		
1	1.95	1.39–2.75	**<0.001**	1.25	0.86–1.81	0.082
2	3.55	2.29–5.51	**<0.001**	1.84	1.13–3.01	**<0.05**
Comorbidity						
Pulmonary disease	1.30	0.82–2.04	0.262	1.10	0.62–1.95	0.735
Renal disease	0.66	0.38–1.15	0.141	2.94	1.64–5.24	**<0.001**
Liver disease	0.91	0.55–1.52	0.720	0.63	0.27–1.44	0.273
T2DM	0.79	0.56–1.11	0.179	0.98	0.59–1.65	0.951
Hyperlipidemia	1.13	0.82–1.57	0.447	0.68	0.42–1.10	0.114

* Adjustment for age, sex, medication, and physical comorbidities. Bold type indicates statistical significance. Abbreviations: CDR, clinical dementia rating scale; CI, confidence interval; HRs, hazard ratios; HTN, hypertension; Ref, reference; T2DM, type 2 diabetic mellitus.

## Data Availability

The data that support the findings of the study are available from the corresponding author upon reasonable request.
